# Parental Preconception and Pre-Hatch Exposure to a Developmental Insult Alters Offspring’s Gene Expression and Epigenetic Regulations: An Avian Model

**DOI:** 10.3390/ijms24055047

**Published:** 2023-03-06

**Authors:** Issam Rimawi, Gadi Turgeman, Nataly Avital-Cohen, Israel Rozenboim, Joseph Yanai

**Affiliations:** 1 The Ross Laboratory for Studies in Neural Birth Defects, Department of Medical Neurobiology, Institute for Medical Research—Israel-Canada, Hadassah Medical School, The Hebrew University, P.O. Box 12272, Jerusalem 91120, Israel; 2 Department of Molecular Biology, Ariel University, Ariel 40700, Israel; 3 Department of Animal Sciences, Robert H. Smith Faculty of Agriculture, Food and Environment, Hebrew University of Jerusalem, P.O. Box 12, Rehovot 76100, Israel; 4 Department of Pharmacology and Cancer Biology, Duke University Medical Center, Durham, NC 27710, USA

**Keywords:** chlorpyrifos, avian model, epigenetic regulation, gene expression, neurogenesis and neurotransmission, parental preconception exposure

## Abstract

Parental exposure to insults was initially considered safe if stopped before conception. In the present investigation, paternal or maternal preconception exposure to the neuroteratogen chlorpyrifos was investigated in a well-controlled avian model (*Fayoumi*) and compared to pre-hatch exposure focusing on molecular alterations. The investigation included the analysis of several neurogenesis, neurotransmission, epigenetic and microRNA genes. A significant decrease in the vesicular acetylcholine transporter (SLC18A3) expression was detected in the female offspring in the three investigated models: paternal (57.7%, *p* < 0.05), maternal (36%, *p* < 0.05) and pre-hatch (35.6%, *p* < 0.05). Paternal exposure to chlorpyrifos also led to a significant increase in brain-derived neurotrophic factor (BDNF) gene expression mainly in the female offspring (27.6%, *p* < 0.005), while its targeting microRNA, miR-10a, was similarly decreased in both female (50.5%, *p* < 0.05) and male (56%, *p* < 0.05) offspring. Doublecortin’s (DCX) targeting microRNA, miR-29a, was decreased in the offspring after maternal preconception exposure to chlorpyrifos (39.8%, *p* < 0.05). Finally, pre-hatch exposure to chlorpyrifos led to a significant increase in protein kinase C beta (PKCß; 44.1%, *p* < 0.05), methyl-CpG-binding domain protein 2 (MBD2; 44%, *p* < 0.01) and 3 (MBD3; 33%, *p* < 0.05) genes expression in the offspring. Although extensive studies are required to establish a mechanism–phenotype relationship, it should be noted that the current investigation does not include phenotype assessment in the offspring.

## 1. Introduction

It has been well established that prenatal/pre-hatch exposure to insults such as drugs, chemicals and pesticides induces behavioral deficits and molecular alterations in the offspring [1,2,3,4]. Understanding the molecular mechanisms by which teratogens exert their deleterious action enabled the reversal of neurobehavioral deficits by various means [5,6,7].

It appears, as a natural subsequent step, to establish a similar model of neurobehavioral teratogenicity for parental preconception exposure. Although first investigated in the early nineteen hundreds [8], consequences of parental preconception insult exposure on the offspring received, until recent years, limited attention. The recent increased interest in maternal and paternal preconception insult exposure effects on the offspring is probably due to a better understanding of the epigenetic mechanisms involved in such exposures (for review, see [9]). DNA methylation, histone modification and non-coding RNA transmission are the main proposed epigenetic mechanisms mediating the transfer of deficits to the offspring after paternal insult exposure (for review, see [10]). Previous studies on parental preconception exposure to insults (mainly performed on rodents) showed that maternal and/or paternal preconception insult exposure could have detrimental effects (demonstrated molecularly, biochemically and/or behaviorally) on the offspring (for review, see [9]). Many insults were investigated in such studies, including chemicals, substances of abuse, therapeutic agents and even lifestyle factors (for review, see [9]).

Organophosphates, including chlorpyrifos, are among the most widely used insecticides worldwide. Chlorpyrifos is a widely studied archetypal neuroteratogen, and extensive information is available on its deleterious action on neurodevelopment [11,12]. Chlorpyrifos works by inhibiting the activity of the enzyme acetylcholinesterase (AChE) [13]. Cholinesterase inhibition following chlorpyrifos exposure can persist for weeks [14]; consequently, frequent exposure to small amounts of chlorpyrifos might cause acetylcholine accumulation and sudden-onset acute toxicity [14]. Metabolic activation of chlorpyrifos is mediated by cytochrome P450 (CYP450) oxidative desulfurization of the P=S moiety to P=O moiety, resulting in the toxic metabolite chlorpyrifos oxon [15,16,17,18]. Chlorpyrifos possesses different stabilities at different pH values, obtaining a half-life of 16 days (pH 9) up to 73 days (pH 5) [19]. The half-life of chlorpyrifos ranges from 2 weeks to 1 year in soil, and it is dependent on many factors, including soil type, pH, climate and other conditions [14].

Although the environmental exposure to chlorpyrifos could occur through different administration routes and at different levels than those applied in the current research (chronic exposure to 12.2 mg/kg in chicken feed throughout chickens’ lives [20]), the environmental effects of chlorpyrifos on chickens is not relevant to the current investigation, and chlorpyrifos was used here as a model developmental insult to study the neuroteratogenic effects of parental preconception exposure on the offspring.

Neurobehavioral alterations in the offspring after prenatal exposure to neuroteratogens such as alcohol, heroin and chlorpyrifos is usually accompanied with gene expression changes. Alterations in normal control of gene expression and reprogramming during embryonic development typically occur following prenatal alcohol exposure, ultimately leading to fetal alcohol syndrome-accompanied behavioral alterations [21]. Neurobehavioral alterations after prenatal heroin exposure are well documented [22], and these alterations can be accompanied by several gene expression changes, as shown in mouse models [23]. Fetal exposure to chlorpyrifos in mice and humans causes neurobehavioral alterations that could also be accompanied by gene expression alterations [24,25].

In the avian model, neuroteratogens can be administered in defined doses with little consideration for the pregnancy stage-related pharmacokinetic changes, maternal–fetal interactions or maternal toxicities that are not directly related to the neuroteratogen exposure, since the egg is situated outside of the neuroteratogen-exposed mother from an early developmental stage [26].

Most studies on avian models for neurobehavioral teratology apply broilers or layer strains (for review, see [27]). For preconception exposure studies, it is advantageous to use robust strains that could survive severe teratogen exposure at maturity, during mating and egg laying. The strain used in the current investigation, *Fayoumi*, is usually exposed to harsh conditions (for review, see [28]) while being classified as a relatively resistant strain to most severe poultry diseases [29]. Our preliminary studies suggest that this strain survives preconception exposure to chlorpyrifos much better than the highly sensitive broiler strains and even better than the commercial layer strains, especially in situations where chickens are exposed to the neuroteratogen for extended periods (unpublished data).

Our previous studies demonstrated neurogenesis and neurotransmission genes expression alterations in the offspring following pre-hatch exposure to chlorpyrifos [3,30]. Additional studies investigating the effects of prenatal/pre-hatch exposure to chlorpyrifos observed several signaling cascades deficits, epigenetic alterations and/or genes expression modifications in the offspring in animal [31,32,33,34] and human models [24]. In addition to their direct toxic effects, prenatal exposure to neuroteratogens could inflict deleterious actions on the offspring’s neurobehavioral development indirectly, for example, leading to cell death [35,36] and perturbing neural circuitries [37,38]. Several studies performed on rodents confirmed that parental preconception exposure to neuroteratogens could also affect signaling cascades [39,40], epigenetic regulations [41] and gene expression [42,43].

Our recent review of parental preconception exposure to insults [9] indicates that the effects of preconception exposure (especially paternal) to insults on the offspring seem to be directly related to changes in gene expression mediated by epigenetic regulation mechanisms. This observation, along with our previous observations that chlorpyrifos affects neurogenesis and neurotransmission genes expression [3,30], provided the rationale to our hypothesis that parental preconception exposure to chlorpyrifos alters neurogenesis and neurotransmission genes expression in the offspring via epigenetic mechanisms. Chlorpyrifos was selected here due to its value for neurobehavioral teratology research (as a neuroteratogen) and not due to its environmental effects on chickens. We considered neuronal deficits in the offspring after parental preconception exposure to chlorpyrifos as preconception neuroteratology.

In the present study, female or male *Fayoumi* chickens were subjected to chlorpyrifos preconceptionally, beginning three weeks prior to eggs collection. The pertinent genes were analyzed in the embryo right before hatching (incubation day 20). To control for possible direct chlorpyrifos exposure of the early embryo, chlorpyrifos was analyzed in the maternally exposed eggs. The gene analysis results in the preconception model were compared to those obtained from the pre-hatch model, where embryos were exposed to chlorpyrifos on incubation days 0 and 5.

## 2. Results

### 2.1. Sex Determination

The developed chromodomain helicase DNA-binding Z and W (CHDZ and CHDW) primers accurately differentiated between genders in 75 out of 76 samples. RT-PCR analysis using SYBR green dye demonstrated undetected CHDW expression in male samples (Ct > 35), while it was distinctly detected in female samples (Ct < 25; Supp. S1, Figure S1). CHDZ was used as a housekeeping gene, and its expression was detected in both sexes.

### 2.2. Paternal Exposure to Chlorpyrifos Alters Gene Expression in Offspring

**Figure 1:**
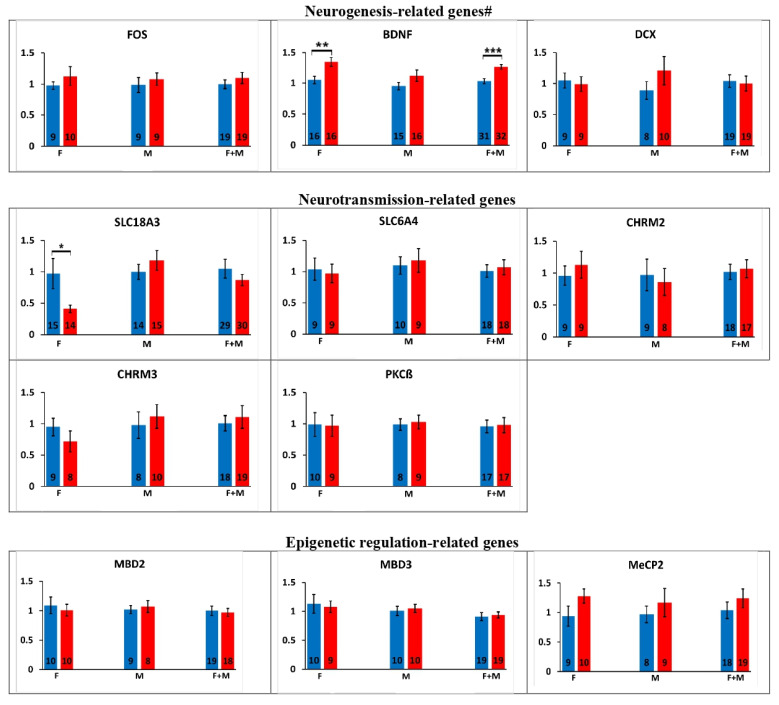

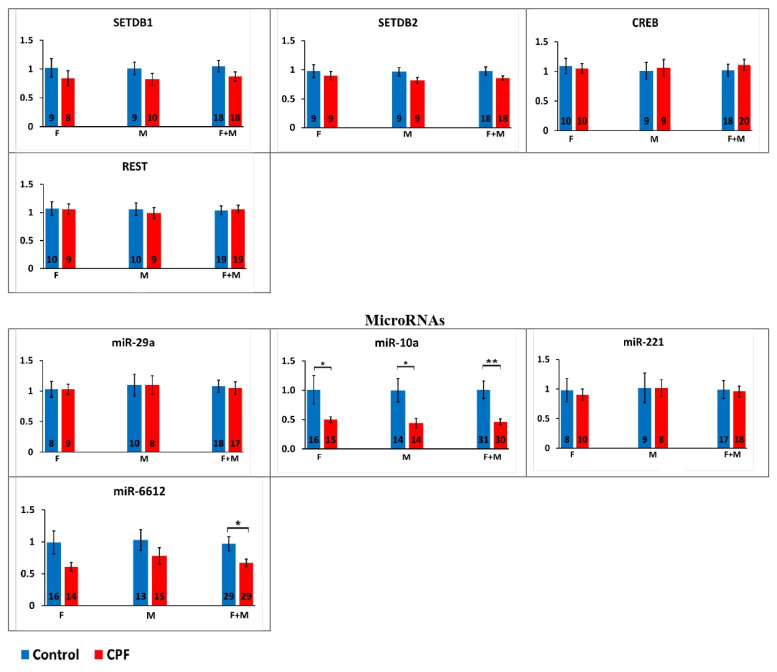
Effects of paternal exposure to chlorpyrifos (CPF) on the offspring’s gene expression. Relative gene expression results obtained in the offspring after paternal exposure to chlorpyrifos. M: male offspring, F: female offspring. Number of samples (n) is presented inside each column. Results are presented as the mean ± SEM. #: PKCß, which is related to both neurogenesis and neurotransmission genes, is presented in the neurotransmission section. *: *p* < 0.05, **: *p* < 0.005 and ***: *p* < 0.0005. PKCß: protein kinase C beta, BDNF: brain-derived neurotrophic factor, FOS: C-Fos, DCX: doublecortin, CHRM2 and CHRM3: muscarinic receptors 2 and 3, SLC18A3: solute carrier family 18 member A3, SLC6A4: solute carrier family 6 member 4, MeCP2: methyl CpG binding protein 2, MBD2 and MBD3: methyl-CpG-binding domain proteins 2 and 3, SETDB1 and SETDB2: SET domain bifurcated histone lysine methyltransferase 1 and 2, CREB: cAMP-response element binding protein, REST: RE1 silencing transcription factor, miR-221: microRNA 221, miR-29a: microRNA 29a, miR-6612: microRNA 6612 and miR-10a: microRNA 10a.

Alterations in the offspring’s gene expression after paternal exposure to chlorpyrifos are shown in Figure 1.

Neurogenesis-related genes: Paternal exposure to chlorpyrifos resulted in significant increase in brain-derived neurotrophic factor (BDNF) gene expression (22.3%, *p* < 0.0005). Treatment by sex analysis suggested that female offspring were the main contributors to this significance (27.6%, *p* < 0.005). BDNF expression in the male offspring was less impacted (non-significant increase) but still contributed to the overall significance, since the treatment by sex interaction did not exist (*p* > 0.38). No additional significant alterations were observed in the expression of doublecortin (DCX) or C-Fos (FOS) in the paternally exposed offspring.

Neurotransmission-related genes: Paternal exposure to chlorpyrifos led to a significant decrease in the vesicular acetylcholine transporter (solute carrier family 18 member A3; SLC18A3) expression only in the female offspring (57.7%, *p* < 0.05). Gene expression of protein kinase C beta (PKCß), the cholinergic muscarinic receptors 2 and 3 (CHRM2 and CHRM3), and of the serotonergic transporter (solute carrier family 6 member 4; SLC6A4) was not affected significantly in the offspring after paternal exposure to chlorpyrifos.

Epigenetic regulation-related genes: No significant alteration was observed in the epigenetic regulations-related genes (methyl-CpG-binding domain proteins 2 and 3 (MBD2 and MBD3), methyl CpG binding protein 2 (MeCP2), SET domain bifurcated histone lysine methyltransferase 1 and 2 (SETDB1 and SETDB2), cAMP-response element binding protein (CREB) and RE1 silencing transcription factor (REST)) expression following paternal exposure to chlorpyrifos.

microRNA genes: BDNF’s targeting microRNA, microRNA 10a (miR-10a) [44], was decreased in the paternally exposed offspring (54.5%, *p* < 0.005). microRNA 6612 (miR-6612), which targets PKCß’s mRNA [45], was also reduced in the exposed offspring (30.9%, *p* < 0.05). microRNA 221 (miR-221) and microRNA 29a (miR-29a) gene expression was not significantly altered in the offspring after paternal exposure to chlorpyrifos.

### 2.3. Maternal Preconception Exposure to Chlorpyrifos Alters Gene Expression in the Offspring

#### 2.3.1. Gene Expression Alterations

**Figure 2:**
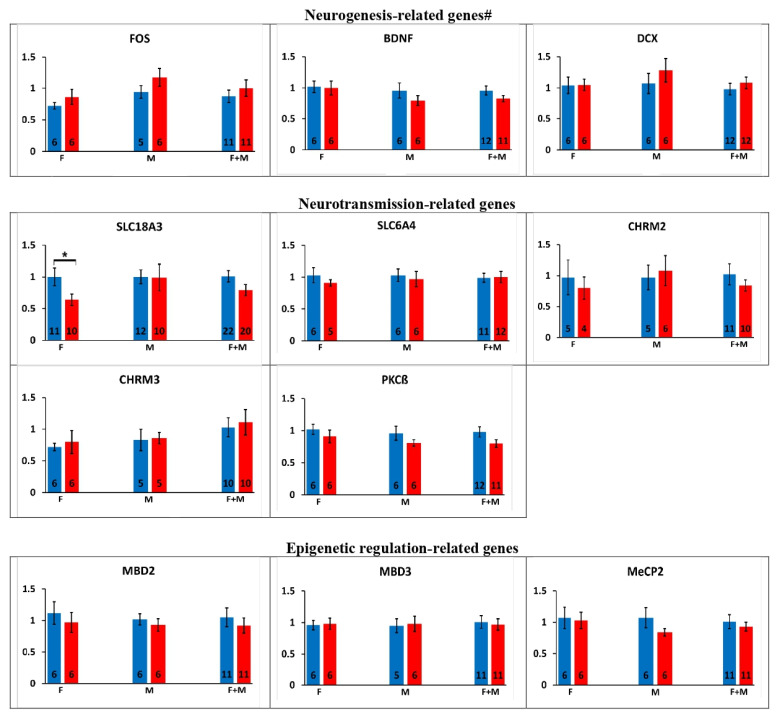

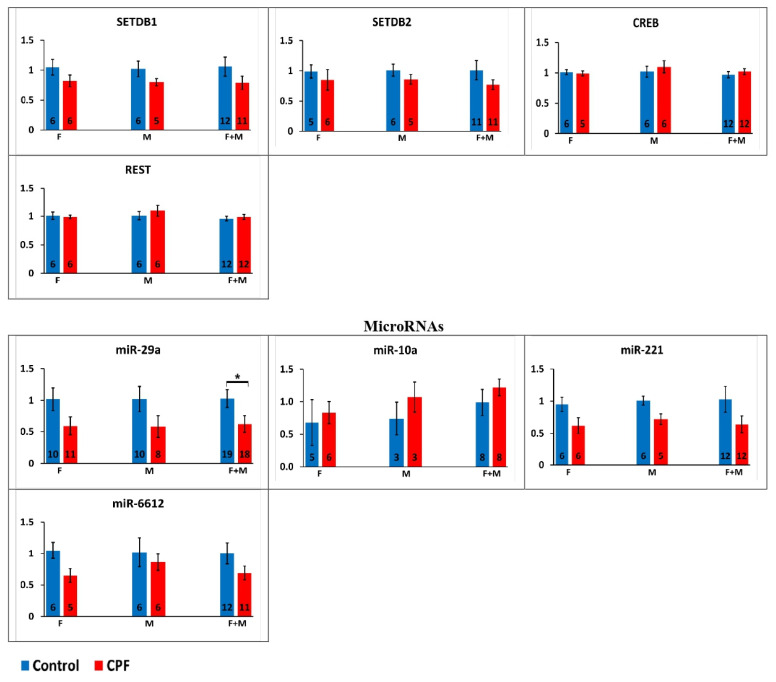
Effects of maternal preconception exposure to chlorpyrifos (CPF) on the offspring’s gene expression. Relative gene expression results obtained in the offspring after pre-hatch exposure to chlorpyrifos. M: male offspring, F: female offspring. Number of samples (n) is presented inside each column. Results are presented as the mean ± SEM. #: PKCß, which is related to both neurogenesis and neurotransmission genes, is presented in the neurotransmission section. *: *p* < 0.05. For genes abbreviations, please refer to Figure 1.

Alterations in the offspring’s gene expression after maternal preconception exposure to chlorpyrifos are shown in Figure 2.

Neurogenesis-related genes: No significant alterations were observed in the offspring’s neurogenesis-related genes expression after maternal preconception exposure to chlorpyrifos.

Neurotransmission-related genes: Similar to the results obtained after paternal exposure, maternal preconception exposure to chlorpyrifos caused a significant decrease in the cholinergic transporter (SLC18A3) gene expression only in the female offspring (36%, *p* < 0.05). No additional significant alterations were observed in PKCß, the cholinergic muscarinic receptors 2 and 3 or in the serotonergic transporter (SLC6A4) genes expression.

Epigenetic regulation-related genes: No significant alteration was observed in the epigenetic regulation-related genes (MeCP2, MBD2, MBD3, SETDB1, SETDB2, CREB and REST) expression after maternal preconception exposure to chlorpyrifos.

microRNA genes: DCX’s targeting microRNA, miR-29a [46], was decreased in the offspring after maternal preconception exposure to chlorpyrifos (39.8%, *p* < 0.05). miR-221, miR-10a and miR-6612 genes expression was not altered significantly in the exposed offspring.

#### 2.3.2. Chlorpyrifos Residues

Residues of chlorpyrifos were undetected in the control eggs (n = 2), while, in the maternal preconception-exposed eggs, chlorpyrifos residues were detected (2.792–3.253 mg/kg; n = 2). Residues of chlorpyrifos methyl and chlorpyrifos’s active metabolite, chlorpyrifos oxon, were undetected in all samples.

### 2.4. Pre-Hatch Exposure to Chlorpyrifos Alters Gene Expression in the Offspring

**Figure 3:**
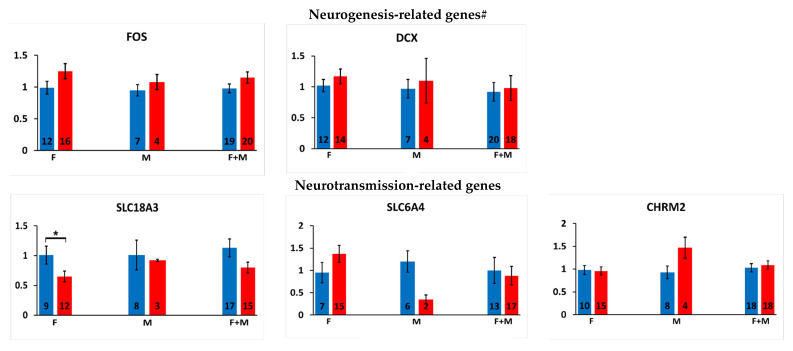

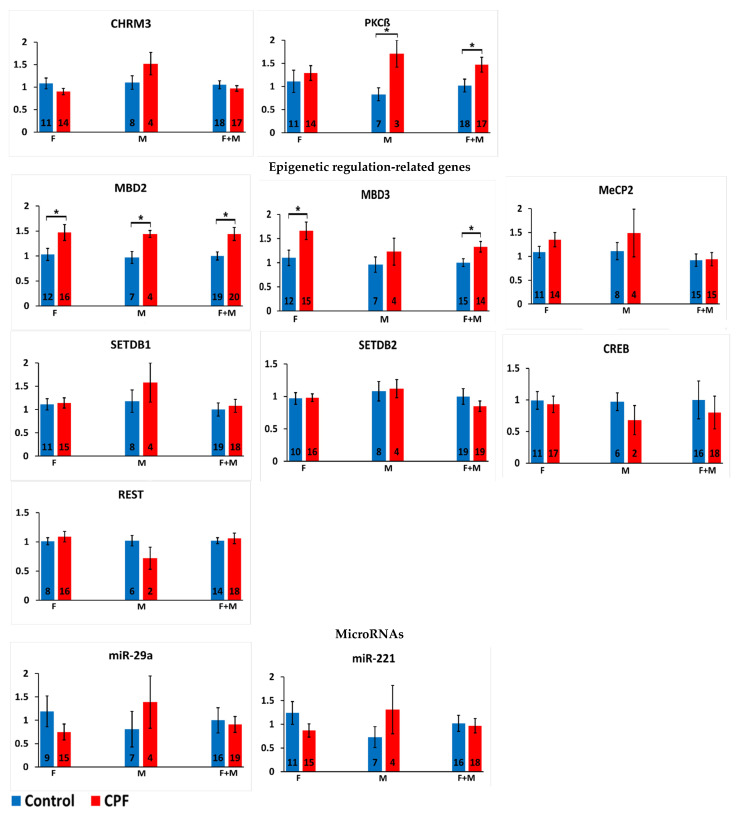
Effects of pre-hatch exposure to chlorpyrifos (CPF) on the offspring’s gene expression. Relative gene expression results obtained in the offspring after pre-hatch exposure to chlorpyrifos. M: male offspring, F: female offspring. Number of samples (n) is presented inside each column. Results are presented as the mean ± SEM. #: PKCß, which is related to both neurogenesis and neutransmission genes, is presented in the neurotransmission section. *: *p* < 0.05. For genes abbreviations, please refer to Figure 1.

Alterations in the offspring’s gene expression after pre-hatch exposure to chlorpyrifos are shown in Figure 3.

Neurogenesis-related genes: DCX and FOS gene expression was not altered significantly in the offspring exposed to pre-hatch chlorpyrifos.

Neurotransmission-related genes: Pre-hatch exposure to chlorpyrifos led to a significant increase in PKCß expression in the offspring (44.1%, *p* < 0.05). This alteration was mainly contributed by the male offspring (females showed nonspecific increase), and treatment by sex interaction analysis was not significant (*p* > 0.35). Female offspring showed a significant decrease in the cholinergic transporter (SLC18A3) gene expression (35.6%, *p* < 0.05). Gene expression of the cholinergic muscarinic receptors 2 and 3 and of the serotonergic transporter, SLC6A4,was not affected significantly in the offspring after pre-hatch exposure to chlorpyrifos.

Epigenetic regulation-related genes: Offspring exposed to pre-hatch chlorpyrifos showed a significant increase in the DNA methylation readers MBD2 (44%, *p* < 0.01) and MBD3 (33%, *p* < 0.05; mainly contributed by the female offspring) gene expression. The rest of the epigenetic regulation-related genes (MeCP2, SETDB1, SETDB2, CREB and REST) were not affected significantly in the offspring after pre-hatch exposure to chlorpyrifos.

microRNA genes: miR-221 and miR-29a gene expression was not altered significantly in the offspring after pre-hatch exposure to chlorpyrifos.

### 2.5. Gene Co-Expression Correlation Network Analysis Reveals Disrupted Regulatory Network in Chlorpyrifos Affected Offspring

**Figure 4:**
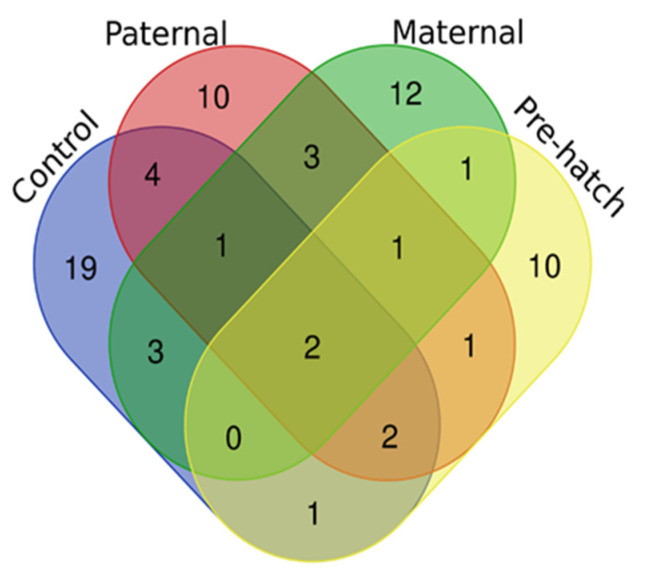
Venn diagram representing shared and non-shared gene expression correlation pairs in the control and chlorpyrifos exposed (paternally, maternally and pre-hatch) offspring.

To better understand the chlorpyrifos exposure effects on possible interaction mechanisms between the investigated genes, we performed a correlation matrix analysis. Statistically significant correlations (Spearman’s *p* < 0.05) were considered. The control offspring displayed 32 correlations between the tested genes (Supp. S2). Paternal chlorpyrifos exposure offspring shared with the control offspring nine of these correlations, while maternal and pre-hatch offspring shared six and five, respectively (Figure 4). Only two correlations were shared among all offspring, namely positive correlations between REST and CREB and between SETDB1 and SETDB2 (Supp. S2–S5).

**Figure 5:**
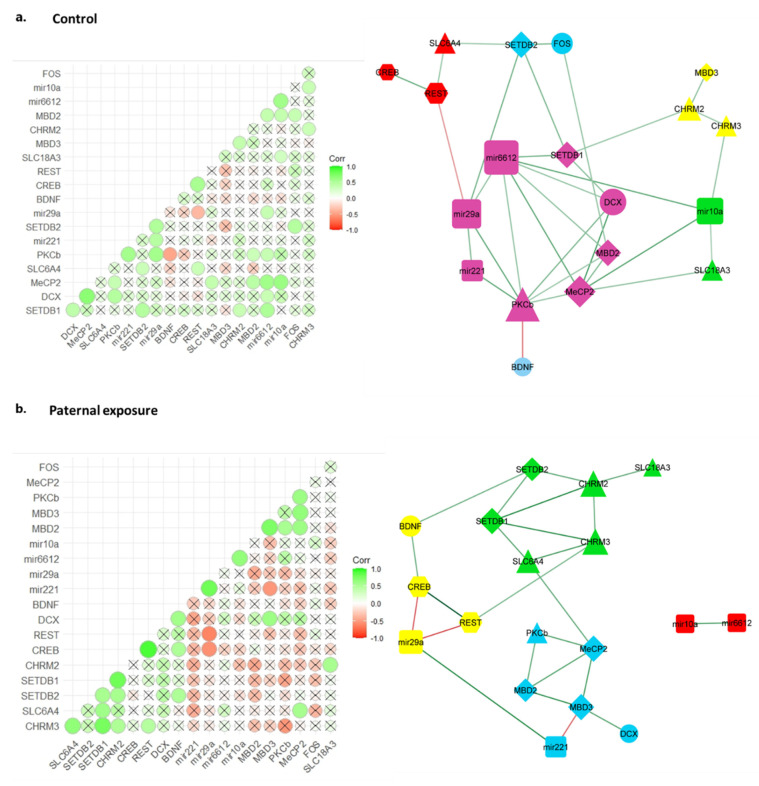

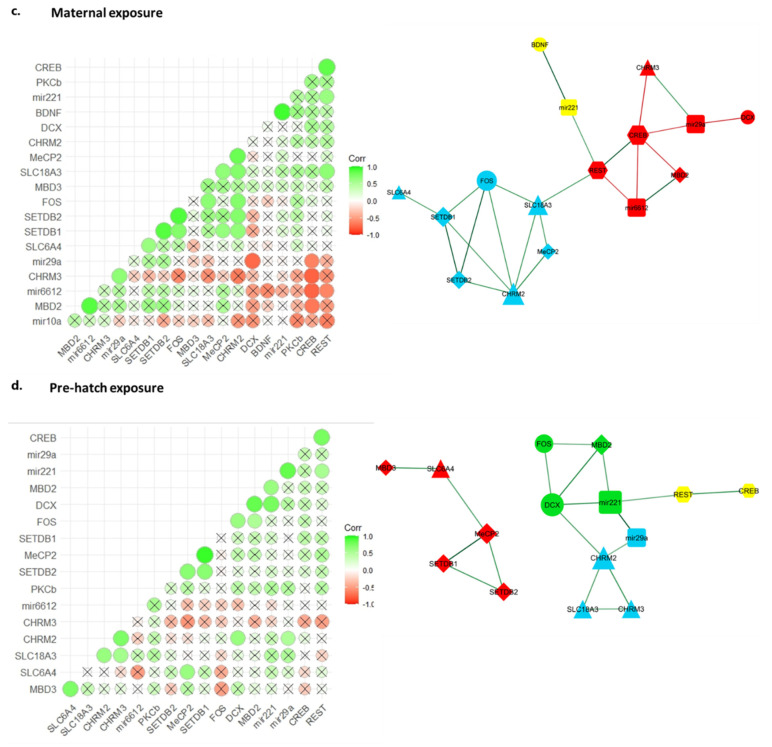
Gene co-expression correlation matrix and network in the offspring of the control (**a**), paternally (**b**), maternally (**c**), and pre-hatch (**d**) chlorpyrifos-exposed chickens. In the left panel, a correlation matrix between the different genes. Non-statistically significant correlations are marked in x. In the right panel, correlation networks between the genes represented as nodes and their correlation as edges. Node size represents the number of connecting edges of the network; only statistically significant correlations were considered. Detected modules (nodes communities) are stained with different colors. Module 1—red, Module 2—yellow, Module 3—light blue, Module 4—green and Module 5—purple. In both panels, edges and correlation color intensity signifies an increased correlation, with green for positive correlations and red for negative correlations.

Correlation matrices were used to generate unweighted gene co-expression correlation networks. The control offspring presented a 19-node correlation network with a main node module composed of eight genes, including three microRNAs (miR-6612, miR-221 and miR-29a); the chromatin modifier (SETDB1); two methylated DNA readers (MECP2 and MDB2); the neurogenesis-related gene (DCX) and the neurotransmission-related gene (PKCß). The hub genes in this module were miR-6612 and PKCß, which interacted with six of the seven remaining nodes (Figure 5a).

Interestingly, chlorpyrifos-exposed chickens presented less complex networks and modules, with a reduced number of edges compared to the controls. All chlorpyrifos-exposed animals showed a reduced involvement of microRNAs and, in particular, that of miR-6612.

In paternally exposed offspring, two central six-node modules were detected. Module 3 corresponded to the main module in the controls (module 5) and shared four nodes with it (DCX, PKCß, miR-221 and MeCP2), with MDB3 replacing MDB2. Module 4 connected all neurotransmitter-related genes with the chromatin modifiers (SETDB1 and SETDB2) (Figure 5b). Among all correlations found in paternally exposed offspring, only 5 out of 24 involved microRNAs, a rate significantly lower (*X*^2^, *p* < 0.05) than that observed in the controls (15 of 32).

In maternally exposed offspring, module 3 was the dominant module, with seven nodes and 11 edges. Module 3 resembled module 4 in parentally exposed offspring containing the neurotransmitter-associated genes together with the chromatin modifiers (SETDB1 and SETDB2), in addition to MeCP2 and FOS (Figure 5c). Module 1, having seven nodes, shared four nodes (miR-6610, miR-29a, DCX and MDB2) with the main module of the control offspring network (module 5). However, the number of edges and connectivity in this module were tremendously altered. Six of the nine correlations in this module were negative correlations. miR-6612 presented three correlations, two of which were negative correlations with CREB and REST and only one positive correlation with MDB2 (the only correlation in this module resembling control correlations).

In the offspring exposed to pre-hatch chlorpyrifos, a further reduced complexity of the network was observed (Figure 5d). The largest module had only five nodes and five edges and included the chromatin modifiers SETDB1 and SETDB2 and the methylated DNA readers MDB3 and MeCP2, together with the serotonergic gene SLC6A4. Module 4 shared three of its four nodes (DCX, MDB2 and MeCP2) with the main module of the control network but shred none of the edges connecting the nodes.

### 2.6. Egg and Body Weights of the Offspring

Egg weights and offspring’s body weights of pre-hatch, maternal and paternal exposures are presented in Table 1. Significant weight alterations were observed only after maternal exposure to chlorpyrifos with an increase in egg weights (11.9%; *p* < 0.005) and in the offspring’s body weights (5.1%; *p* < 0.05).

## 3. Discussion

The current investigation indicated that exposure to chlorpyrifos, a significant neuroteratogen, could have deleterious effects on the offspring, even if the exposure occurred prior to pregnancy. In the current study, parental preconception exposure to chlorpyrifos in an avian model altered the expression of neurogenesis and neurotransmission-related genes, as well as the regulating epigenetic genes. The results were compared to alterations in gene expression induced by pre-hatch exposure to chlorpyrifos, and they did not necessarily correspond.

Our previous work extensively studied the effects of early (prenatal (mice)/pre-hatch (chick)) exposure to several cholinergic and cholinergic-related teratogens (chlorpyrifos, heroin and nicotine) on the offspring’s behavior and the mechanistically related changes in neurotransmission and neurogenesis converging into the abolishment of cholinergic receptor-induced activation/translocation of PKC isoforms [3,47,48,49,50]. In the present research, we extend the study to another form of early exposure, parental exposure, and investigated the alterations in neurogenesis and neurotransmission gene expression as related to the previously observed behavioral deficits.

It has been previously established that prenatal (mice)/pre-hatch (chicks) exposure to neuroteratogens, including chlorpyrifos, induces significant deficits in learning-related behaviors in the offspring (seen as imprinting behavior deficits in our previous pre-hatch model [3]) and that these deficits are the outcome of alterations in neurogenesis and neurotransmission-related genes, particularly the cholinergic receptor-mediated abolishment of activation/translocation of PKC isoforms [3,51]. Activation/translocation of PKC isoforms was abolished after introducing IMM tissues (extracted from offspring after pre-hatch exposure to chlorpyrifos) to the cholinergic agonist, carbachol, which was correlated with the imprinting behavior deficits seen in the same offspring [3].

We hypothesized that parental preconception exposure to the neuroteratogen chlorpyrifos would affect the offspring’s neurogenesis and neurotransmission genes expression and that those effects would be mediated by epigenetic regulation mechanisms. Consequently, we analyzed the expression of neurogenesis (BDNF, PKCß, DCX and FOS) and neurotransmission (CHRM2, CHRM3, SLC6A4 and SLC18A3) genes and microRNA genes (miR-221, miR-29a, miR-6612 and miR-10a), along with several epigenetic genes (MeCP2, MDB2, MBD3, SETDB1, SETDB2, REST and CREB), most of which were previously linked to neuronal functions.

The neurogenesis-related genes included BDNF (a crucial neurotrophin involved in plastic alterations related to learning and memory [52]), DCX (serves as an efficient marker for neurogenesis [53,54,55]), PKCß (phosphorylation of PKC substrates may be involved in neuronal plasticity and growth [56]) and FOS (can be used as marker for neuronal activity following deleterious stimulation and tissue injury [57,58,59,60]). These genes’ expression was previously shown to be affected by prenatal/pre-hatch or direct exposure to chlorpyrifos in several studies, including our own [3,30,31,32,33], suggesting that the same genes might be affected in the offspring after paternal/maternal preconception exposure to chlorpyrifos. Indeed, in the current study, paternal preconception exposure to chlorpyrifos altered the expression of BDNF, suggesting neurogenesis as a mechanism mediating functional deficits in the offspring after paternal exposure to insults.

Our previous studies indicate that both serotonergic and cholinergic innervations are affected in the offspring following pre-hatch exposure to chlorpyrifos [3,30]. The investigated cholinergic and serotonergic genes included SLC6A4 (the principal regulator of serotonergic neurotransmission [61,62]), SLC18A3 (mediates the transfer of acetylcholine from the cytoplasm into synaptic vesicles [63]), the muscarinic receptors 2 and 3 (CHRM2 and CHRM3) and PKCß (a key enzyme for signal transduction [64]). In the current study, pre-hatch and preconception exposure to chlorpyrifos significantly affected the expression of the cholinergic transporter, SLC18A3, in the offspring. These results, along with our previous finding that pre-hatch chlorpyrifos exposure abolishes the cholinergic activation/translocation of PKC isoforms in the offspring [3], probably indicate that the cholinergic innervation is a key mechanism involved in chlorpyrifos’s neuroteratogenic effects in both pre-hatch and preconception exposure models.

microRNA was investigated as a possible mechanism that might mediate gene expression alterations in the offspring after parental/pre-hatch exposure to chlorpyrifos. The investigated microRNAs included miR-10a (targets BDNF’s mRNA [44]), miR-221 (targets FOS’s mRNA [65]), miR-29a (targets DCX’s mRNA [46]) and miR-6612 (targets PKCß’s mRNA). The selection of the current microRNAs was based on a peer-reviewed online database (TargetScan database), which identifies the targeting microRNAs based on the inserted gene and species [45]. In addition, most of these microRNAs were validated in previous investigations [44,46,65]. The microRNA investigation after parental preconception exposure to insults received limited attention, and only a few studies [66] considered investigating such exposure effects on the offspring’s microRNAs, making our study a pioneering study in this field. microRNA involvement in mediating gene expression alterations in the offspring was mainly observed in the paternal group, where miR-10a was downregulated in the offspring while its target mRNA (BDNF) was upregulated. On the other hand, the gene expression of paternal miR-6612 and maternal miR-29a was altered in the offspring, while their target mRNA (PKCß and DCX, respectively) gene expression was not affected. This is probably due to the fact that microRNAs can bind several target mRNAs, and a single mRNA can be targeted by more than one microRNA [67,68].

Other epigenetic genes that might have mediated chlorpyrifos exposure effects to the offspring were investigated. Those genes included MBD genes (MeCP2, MBD2 and MBD3) and histone modification genes (SETDB1 and SETDB2), which were previously linked to neuronal functions [69,70]. In addition, REST and CREB gene expression was investigated. REST binds the neuron-restrictive silencer element that represses neuronal gene transcription in nonneuronal cells [71,72] and is linked to neuronal inflammation [73]). CREB binds to DNA sequences called cAMP response elements, thereby increasing or decreasing the transcription of downstream genes [74], and its activity in neurons is correlated with various intracellular processes, including proliferation, differentiation, survival, long-term synaptic potentiation, neurogenesis and neuronal plasticity [75,76,77].

Although some of the epigenetic genes did not show differential expression in chlorpyrifos-exposed offspring, we estimated their potential regulatory role by performing a co-expression analysis with the other genes. Correlation matrices and the co-expression network showed complex interactions and co-expressions between several microRNAs (miR-6612, miR-221 and miR-29a); the chromatin modifier (SETDB1); methylated DNA readers (MDB2 and MeCP2) and the neurogenesis-related genes (DCX and PKCß) (Figure 5). The involvement of miR-6612 was mainly noticed in the control offspring network. On the other hand, chlorpyrifos-exposed offspring presented disrupted networks, especially regarding the involvement of microRNAs (Figure 5). miR-6612 involvement in gene expression correlations was absent in paternally exposed offspring and was altered in maternally exposed offspring. In addition, miR-6612 and miR-29a were downregulated in the paternally and maternally exposed offspring, respectively, suggesting that alterations in microRNA expression may be the key mechanism mediating the disruption of offspring neurogenesis and neurodevelopment by parental preconception exposure to chlorpyrifos. Indeed, microRNAs are the leading candidates for parental and, especially, paternal epigenetic inheritance via the gametes. Most cytoplasm and RNA contents are ejected from the spermatozoa during the final stages of spermatogenesis. However, several small non-coding RNAs (sncRNAs) and a few mRNAs have been shown to remain in sperm and may enter the oocyte upon fertilization [78,79,80,81,82,83,84,85]. Those sncRNAs were shown to be transferred to the spermatozoa in the epididymis via lipid-rich exosomes called epididymosomes [86,87], and they include microRNAs, endogenous short interfering RNAs and PIWI-interacting RNAs [79,88,89].

This study is one of only a few [41,90] that has considered investigating alterations in the offspring’s epigenetic genes after parental preconception exposure to insults. In the current investigation, MBD2 and MBD3 seemed to play a role in gene expression alterations observed in the offspring following pre-hatch exposure to chlorpyrifos. On the other hand, microRNA seemed to be the primary epigenetic regulator (among those investigated) that mediates gene expression alterations in the offspring after paternal exposure to chlorpyrifos. Further studies are required to investigate the possibility of microRNA transmission to the offspring through sperm cells.

The duration of paternal exposure to chlorpyrifos was selected based on previous studies, which indicates that the spermatogenesis duration is approximately 14 days in most avian models, including the chicken model [91,92,93,94], and, consequently, was intended to affect sperm cells. Accordingly, we exposed the males to chlorpyrifos for two weeks before mating with females.

The methodological obstacles that may arise upon developing a preconception maternal model might have led to the scarcity in the studies investigating this model (for review, see [9]). One of those obstacles in rodents is the possible accumulation of the exposed material/s (e.g., heavy metals from cigarette smoke) in the endometrium [95], which might affect the embryo prenatally, even if the insult was stopped prior to conception. On the other hand, chick embryos mostly develop outside their mothers’ bodies and only the very early stages of their development are spent in the uterus [26]. This implies that the possibility of the early embryos being directly exposed to an insult following maternal preconception exposure can be mostly controlled. In order to validate the integrity of the maternal preconception exposure model and inspect the possibility of chlorpyrifos being carried over to the embryos following this exposure, we performed a quantitative analysis of chlorpyrifos and its active metabolite, chlorpyrifos oxon, in maternal preconception-exposed eggs and compared them to the control eggs. The results were intended to verify whether the effects obtained in the offspring following maternal preconception exposure were obtained due to direct exposure to chlorpyrifos. This is the first study, performed in an avian model, which evaluates possible effects of neuroteratogen transfer to the egg on the offspring’s gene expression after maternal preconception exposure.

The current study showed that only a small portion of chlorpyrifos was carried over to the egg after maternal preconception exposure. Based on our preliminary (unpublished) dose–response evaluations, this portion (≈3 mg/kg) is not enough to induce neurobehavioral alterations in the offspring. The fact that chlorpyrifos has long elimination half-lives in humans (≈27 h [96]) and mice (≈21 h [97]) suggests that it possesses a similar pharmacokinetic profile in chickens. That being said, the detected amount of chlorpyrifos residues (≈3 mg/kg) is far less than the overall amount that probably accumulated in the mothers’ systems after receiving 10 mg/kg/day chlorpyrifos for three weeks. In addition, gene expression alterations in the offspring differ between pre-hatch and maternal preconception exposures, since MBD2, MBD3 and PKCß genes expression was altered only in the pre-hatch-exposed offspring, while miR-29a gene expression was altered only in the maternally exposed offspring. These findings indicate that the alterations in gene expression observed in the offspring after maternal preconception exposure to chlorpyrifos were mainly mediated by an epigenetic inheritance mechanism and are less related to direct embryo exposure to chlorpyrifos. Accordingly, the possibility of chlorpyrifos being transferred to the embryos through the uterus after maternal exposure was greatly minimized in the currently used avian model compared to the rodent model.

It should be reemphasized that the current study is not an environmental toxicology research; rather, it is a basic neurodevelopmental study designed to understand the effects of parental preconception exposure to neuroteratogens on the offspring and compare that to the effects obtained in the offspring after pre-hatch exposure.

In the current research, we considered gender as a factor that might affect the results obtained in the parentally/pre-hatch-exposed offspring. Indeed, female offspring seemed to be more affected by exposure to chlorpyrifos than male offspring. This was mainly seen in gene expression of the cholinergic transporters SLC18A3 (pre-hatch, maternal and paternal exposures) and BDNF (paternal exposure). Although the gene expression of BDNF’s targeting microRNA, miR-10a, was similarly altered in both genders, the consequence of this alteration on BDNF’s gene expression was less prominent in male offspring, which requires further elaboration. Variations in the sex chromosomes, with the males being homogametic (ZZ) and the females being heterogametic (ZW), have been utilized to develop PCR-based methods intended to differentiate between genders [98]. Chromodomain helicase DNA-binding Z and W (CHDZ and CHDW) genes were previously proven to be effective in sex differentiation in chickens and are considered one of the most commonly used genes for this purpose [99,100,101]. In the current study, we developed CHDZ and CHDW primers that accurately differentiated between genders. This method was used to verify the results obtained after determining the sex by visual examination of the gonads.

Analogous in many ways to the hippocampus and its role in cognitive behaviors in mammals, avian species possess the left IMM, which is mechanistically related to learning and memory [102,103]. Since exposure to chlorpyrifos causes significant deficits in imprinting behavior (learning and memory) [3], the left IMM was used for mRNA extraction and analysis in the current investigation.

Chick embryos were sacrificed on incubation day 20, which might decrease the possible confounding factors that could affect each offspring differently (food intake, hatching day and time spent in incubator after hatching). We wished to study the effects of parental chlorpyrifos exposure on developing offspring that were all raised in a similar and well-controlled environment (with minimum environmental bias).

Parental exposure did not cause a reduction in egg weights or in the offspring’s body weights, which, if occurred, could have suggested an indirect effect on the offspring. In fact, maternal preconception exposure to chlorpyrifos caused a slight increase in body weights, which does not seem to be biologically significant.

Further experiments including high-throughput analysis, protein expression, immunofluorescence, Western blot and the regulation of signaling pathways are pertinent to the current research and should be considered in future research. Although adding a group in which both parents are exposed to chlorpyrifos may complicate the identification of the mechanisms through which each parent mediates the deficits, this may be considered in future research.

The biological relevance of using a non-physiological route of administration and of the lack of phenotype studies remains an open question that should be fulfilled in future experiments.

The present research represents a study in “preconception neuroteratology”, which strongly suggests that preconception exposure to chlorpyrifos in a well-controlled avian model affects the offspring’s neurotransmission and neurogenesis genes expression, mainly through regulating epigenetic mechanisms. The next step might include using techniques such as mRNA and small RNA sequencing to further investigate the effects of preconception exposure to chlorpyrifos on the offspring. Future studies are required to investigate whether the molecular alterations observed in the present study after preconception exposure to chlorpyrifos can be linked to the expected neurobehavioral deficits in the offspring, particularly learning and memory. Understanding the mechanisms of parentally induced deficits may provide the means for the reversal of these deficits towards future clinical application.

## 4. Materials and Methods

### 4.1. Chicken Housing

Female and male *Fayoumi* chickens were maintained in our animal facilities under standard laboratory conditions, as specified by the Office of Laboratory Animal Welfare (OLAW) of the National Institutes of Health (NIH) [104]. Chickens were 7–10 months old and were divided into separate groups, where group members were replaced frequently to increase genetic variability. To enable the replacement, parents were taken from a flock of the relevant group (pre-hatch, paternal or maternal). There were always 2 males and 5 females in each cage for each replication.

### 4.2. Chlorpyrifos Administration

#### 4.2.1. Pre-Hatch Exposure to Chlorpyrifos

Male and female *Fayoumi* chickens (Gallus gallus domesticus) were employed as parents in all experiments. Sixteen females and nine males were separated into groups (as described in the *Chicken Housing* section) and were used to generate the eggs used in the pre-hatch model. Eggs were collected three times daily for 10 days and stored at 14 °C. Within three days from collection, the eggs were placed in an incubator for 20 days according to the manufacturer’s instructions (50% humidity on incubation days (ID) 0–18 and 60% humidity on ID 18–20, 37.5 °C). Chlorpyrifos was generously supplied by Adama Ltd. and was injected into the chorioallantois end (pointed end) of the eggs, as previously described [3,30]. Briefly, 10 mg chlorpyrifos/kg of egg was dissolved in dimethyl sulfoxide (DMSO, Merck, vehicle volume: 510 μL/kg of eggs) and injected on incubation days 0 (prior to incubation) and 5, the period during which most of the brain structures develop [105,106]. The injection site was covered with correction fluid (white out). Control eggs received equivalent volumes of DMSO. The chlorpyrifos dosage was based on previous studies that involved dose–response evaluations [3] and on our preliminary studies showing that the parameters described here, including dose and injection schedule, are right below the level of embryotoxicity and gross malformations. To illustrate this, we observed congenital malformations in 25% of the exposed offspring (mainly seen as cervical scoliosis and torticollis or as macrocephaly) after administering three doses of chlorpyrifos (10 mg/kg on incubation days 0, 5 and 13) to 8 eggs.

#### 4.2.2. Parental Preconception Exposure to Chlorpyrifos

**Figure 6:**
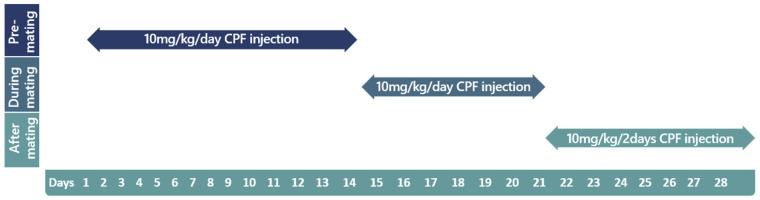
Paternal exposure to chlorpyrifos timeline. Timeline displaying chlorpyrifos exposure doses and periods of male chickens in the paternal exposure group. CPF: chlorpyrifos.

Paternal exposure: Eight male chicken received daily subcutaneous injections of chlorpyrifos (10 mg/kg body weight, dissolved in 300 µL DMSO) at the nape of the neck for 21 days, then received maintenance injections every two days until all eggs were collected (Figure 6). Fourteen days following initial treatment, the exposed males were introduced to 10 untreated females and were separated into groups, as described in the *Chicken Housing* section. Eggs were collected for 10 days (maintenance exposure period), stored at 14 °C and placed in an incubator within 3 days of collection. The respective control groups received equivalent volumes of DMSO and were formed with 8 males and 10 females.

Maternal exposure: The protocol was similar to that of the paternal exposure. Thirteen female chicken received daily subcutaneous injections of chlorpyrifos (10 mg/kg body weight, dissolved in 300 μL DMSO) for 21 days, then received maintenance injections every two days until all eggs were collected. Fourteen days following treatment initiation, 5 untreated males were introduced to the exposed females and were separated into groups, as described in the *Chicken Housing* section. Eggs were collected for 10 days (maintenance exposure period), stored at 14 °C and placed in an incubator within 3 days of collection. The respective control groups received equivalent volumes of DMSO and were formed of 5 males and 13 females.

### 4.3. Tissue Extraction

Chick embryo brains of all groups were removed on ID 20 right before hatching. Laterally extended left intermediate medial hyperstriatum ventrale (IMHV) or intermediate medial mesopallium (IMM), corresponding to the newer nomenclature [107], was extracted according to our modification [3,30] of a previously described procedure [108]. The extracted IMM tissues were stored at −80 °C.

### 4.4. RNA Extraction

Total RNA was extracted separately from the left IMM using an ISOLATE II RNA Mini Kit (Bioline, Memphis, TN, USA). RNA was quantified, and its purity was assessed at an absorbance wavelength of 260 nm using a NanoDrop 2000 spectrophotometer (Thermo Fisher Scientific, Waltham, MA, USA). Extracted RNA was stored at −80 °C.

### 4.5. Real-Time qPCR Analysis

Real-time qPCR analysis was carried out as previously described [109]. Complementary DNA (cDNA) was transcribed from mRNA and microRNA using the qScript cDNA Synthesis Kit (QuantaBio, Beverly, MA, USA) and qScript^®^ microRNA cDNA Synthesis Kit (QuantaBio, MA, USA), respectively. Expression analysis of cDNA samples was performed on the housekeeping genes: glyceraldehyde 3-phosphate dehydrogenase (GAPDH; mRNA analysis) and U6 spliceosomal RNA (RNU6; microRNA analysis); neurotransmission genes: PKCß, CHRM2, CHRM3, SLC18A3 and SLC6A4; neurogenesis genes: BDNF, FOS and DCX; epigenetic related genes: MeCP2, MBD2, MBD3, SETDB1, SETDB2, CREB and REST; sex-determining genes: CHDZ and CHDW; and microRNA genes: miR-221, miR-29a, miR-6612 and miR-10a. microRNAs were selected based on a peer-reviewed online database (TargetScan database), which identifies the targeting microRNAs based on the inserted gene and species (Agarwal et al. 2015). Real-time PCR was carried out using the StepOnePlus and QuantStudio 5 real-time PCR systems (Thermo Fisher Scientific, MA, USA). Genes’ availability in chickens was confirmed using the Gallus gallus genome data viewer (NCBI). Primers were selected, and their specificity was verified using the Primer-BLAST tool (NCBI). The primers were then synthesized commercially (Merck, Rehovot, Israel). A universal reverse microRNA primer was provided in the qScript^®^ microRNA cDNA Synthesis Kit (QuantaBio, MA, USA). Primers used and their sequences are listed in Table 2. Amplification for microRNA genes was performed using PerfeCTa SYBR Green SuperMix (QuantaBio, MA, USA) under the following conditions: preincubation at 95 °C for 2 min, followed by 40 cycles of denaturation at 95 °C for 5 s and annealing at 60 °C for 30 s. Amplification for the rest of the genes was performed using PerfeCTa SYBR Green FastMix ROX (QuantaBio, MA, USA) under the following conditions: preincubation at 95 °C for 20 s, followed by 40 cycles of denaturation at 95 °C for 3 s and annealing at 60 °C for 30 s. microRNA primers’ selectivity was confirmed in multiple minus Poly-A qPCR plate wells that contained treated (with chlorpyrifos) or control samples prepared by the same procedure, excluding the addition of Poly-A tails. Relative quantifications of the target genes were normalized to GAPDH levels in the mRNA analysis and to RNU6 levels in the microRNA analysis and calculated with the 2^−ΔΔCt^ method, as previously described [110]. RT-PCR product specificity was confirmed using a melt curve analysis.

### 4.6. Sex Determination

In the current study, CHDZ and CHDW primers were developed using the Primer BLAST tool (NCBI) to differentiate between chick embryos genders. This method was used to confirm the results obtained after determining the sex by visual examination of the gonads.

### 4.7. Chlorpyrifos Residues

Two control and two maternally exposed eggs (preconception) were collected and homogenized on the same day (eggs were not incubated). Residues of chlorpyrifos, chlorpyrifos methyl and chlorpyrifos oxon in the exposed and control eggs were analyzed using the liquid chromatography-mass spectrometry (LC-MS) technique by the Kimron Veterinary Institute (Beit Dagan, Central District, Israel), as previously described [111]. The detection limit for all tested ingredients was 0.005 mg/kg.

### 4.8. Egg and Body Weights of the Offspring

The possible effect of chlorpyrifos exposure on the offspring’s weights was investigated. Egg and body weights of the embryos were measured on incubation day 20 prior to their brain extraction. The weight means of chlorpyrifos exposed and control samples were calculated and compared for each exposure group (paternal, maternal or pre-hatch) as separate.

### 4.9. Statistical Data Analysis

Multiple-level analysis of variance (ANOVA) was employed for comparing groups. Data were expressed as the mean ± SEM. Genes expression in the offspring exposed to chlorpyrifos (paternal, maternal or pre-hatch model) was compared to the control offspring (not exposed to chlorpyrifos nor were their parents) of the same model. One-way ANOVA was used to test the effects of treatment on chlorpyrifos exposed and control groups where the dependent variable was the gene expression score. Two-way ANOVA included treatment and gender factors (independent variables) in the analysis (since a possible gender effect on the offspring’s genes’ expression was considered) and showed no significant interaction between gender and treatment in any of the tested models (paternal, maternal or pre-hatch). Consequently, after analyzing gene expression alterations in the female and male offspring separately, the results of both offspring were pooled. Post-hoc Tukey’s test was used when appropriate. A correlation analysis was performed using Spearman’s correlation, since part of the data did not show normal distribution, as indicated by the Shapiro–Wilk test. The significance level for all employed tests was considered at *p* < 0.05. Spearman’s correlations between expressed genes were calculated using Prizm GraphPad 9 software. Correlation networks (unsupervised and unweighted) were visualized using Cytoscape software [112], with the Cyfinder application applied for the detection of node communities (modules) according to the edge-betweenness criteria. Correlation matrices were visualized using R packages “ggplot2” and “ggcorrplot”. Venn diagrams were prepared in a designated webtool (https://bioinformatics.psb.ugent.be/webtools/Venn/ (accessed on 3 October 2022)).

## Figures and Tables

**Table 1:** Egg weights and offspring’s body weights after pre-hatch, maternal and paternal exposures.

Exposure	Treatment	#Egg Weight (n)	Body Weight (n)
Pre-hatch	Control	43.6 ± 0.6 (45)	29.5 ± 1.3 (27)
Pre-hatch	CPF	43.9 ± 0.6 (45)	29.8 ± 1.5 (21)
Maternal	Control	35.5 ± 0.9 (45)	20.1 ± 0.3 (46)
Maternal	CPF	39.8 ± 0.8 * (53)	21.2 ± 0.3 * (53)
Paternal	Control	43.8 ± 0.6 (46)	26.2 ± 0.7 (47)
Paternal	CPF	44.2 ± 0.4 (41)	24.4 ± 0.6 (39)

#Egg weights were measured on incubation day 20. Egg and body weights results are in grams and are presented as the mean ± SEM. Number of samples (n) is displayed after each result. * Differences in egg and body weights were significant compared to the control group.

**Table 2:** Sequences of the used primers.

Primer	Sequence (5′-3′)
DCX Forward	AGAAGACGGCCCATTCGTTT
DCX Reverse	GGTCACCTGCTTTCCATCCATCCA
PKCß Forward	CAAACGCCATTTCTAAGTTCGA
PKCß Reverse	CAGCATCACCTTCCCAAAGC
BDNF Forward	AGCAGTCAAGTGCCTTTGGAA
BDNF Reverse	AGTGACGCCGGACTCTCATG
FOS Forward	ACCTCCTCCAGAGATGTAG
FOS Reverse	AGCACCAGTTAATTCCAATC
CHRM2 Forward	TGGAACATAACAAAGTCCAG
CHRM2 Reverse	TTTGTATTGGAAGGAACCAC
CHRM3 Forward	TGGTATATCCAACTACAGGC
CHRM3 Reverse	ACAACTGGTAGTGTGTCAG
SLC18A3 Forward	GATAGACCCCTATATCGCC
SLC18A3 Reverse	CATGGACTCCTTCATCCAG
SLC6A4 Forward	ATAATGCATGGAACACAGG
SLC6A4 Reverse	TGGCGGGTATAAAATTCTTC
CHDZ Forward	AGTCAGGCAGTCAATCCGAA
CHDZ Reverse	CCACTGTCTGATGATGCTGC
CHDW Forward	GGGATTCTGAGTGAAGACTGG
CHDW Reverse	CTGTCCTGTGCCCTTCTTG
MBD2 Forward	CATCCCAATAAGGGCAAAC
MBD2 Reverse	GGGTTGCTTAAAGATGGAC
MBD3 Forward	AAGAAGGAGAAGATGAGGAG
MBD3 Reverse	GAATTCTTAGATGGAACTCCC
MeCP2 Forward	GGACCAGGAAGCTCAAACAG
MeCP2 Reverse	GAAGTACGCGATCAGTTCCAC
SETDB1 Forward	CTCCTTCGTCTGCATCTACG
SETDB1 Reverse	AAGTATTCGTCGCCCATCTC
SETDB2 Forward	AGACAGGCAAAACAAGGCATA
SETDB2 Reverse	AGCAGCTGTGATTAAGAAAACG
REST Forward	AGAGGAAACCAGTCCGAAGA
REST Reverse	CATTGACCAAGTGGCGATT
CREB Forward	GAGAGAATAAAACTGCGGC
CREB Reverse	CCATGGTCATTTAGTTACCG
GAPDH Forward	TGGAGCCCCTGCTCTTCA
GAPDH Reverse	GGAACAGAACTGGCCTCTCACT
miR-221 Forward	AGCTACATTGTCTGCTGGGTTTC
miR-29a Forward	TAGCACCATTTGAAATCGGTT
miR-6612 Forward	ATGCTGTGTGTGCGTTCGTA
miR-10a Forward	TGTGTAAAGGAAGTTGGGTCACA
RNU6 Forward	GCAAATTCGTGAAGCGTTCC
Universal microRNA Reverse Primer	GCATAGACCTGAATGGCGGTA

## Data Availability

The data presented in this study are available in [supplementary material Supp. S1–S5].

## Supplementary Materials

The following supporting information can be downloaded at: https://www.mdpi.com/article/10.3390/ijms24055047/s1.
