# Supplementary material for: Parental Preconception and Pre-Hatch Exposure to a Developmental Insult Alters Offspring’s Gene Expression and Epigenetic Regulations: An Avian Model
Source: Int J Mol Sci. 2023 Mar 6;24(5):5047. doi: 10.3390/ijms24055047 (PMC10003510; doi:10.3390/ijms24055047)
Supplement: Supplementary file 1 [file ijms-24-05047-s001.zip › Supp. S1 - Amplification plot of CHDW.pdf]

Supplementary data 1

Figure S1. Amplification plot of CHDW gene in male (a) and female (b) samples

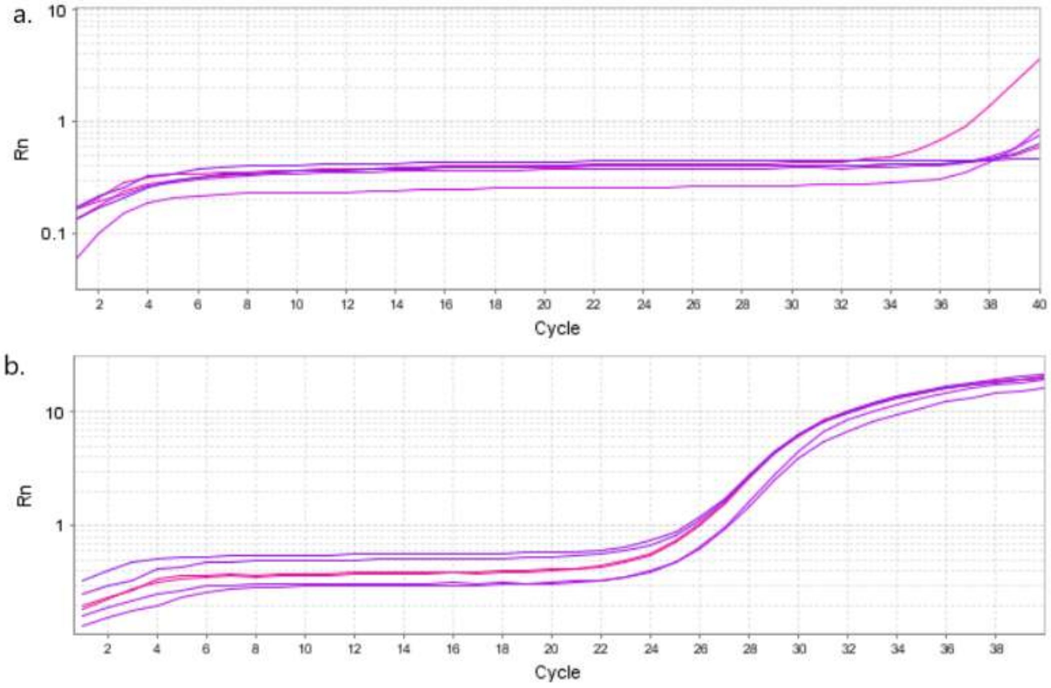

Real-time PCR amplification plot for CHDW gene, showing the normalized reporter (Rn) plotted versus cycle number (Cycle). CHDW gene expression was undetected in male samples (Ct>35) while being distinctly detected in female samples (Ct<25).
